# Human Anti-Plague Monoclonal Antibodies Protect Mice from *Yersinia pestis* in a Bubonic Plague Model

**DOI:** 10.1371/journal.pone.0013047

**Published:** 2010-10-13

**Authors:** Xiaodong Xiao, Zhongyu Zhu, Jennifer L. Dankmeyer, Michael M. Wormald, Randy L. Fast, Patricia L. Worsham, Christopher K. Cote, Kei Amemiya, Dimiter S. Dimitrov

**Affiliations:** 1 Protein Interactions Group, Center for Cancer Research Nanobiology Program, National Cancer Institute-Frederick, National Institutes of Health, Frederick, Maryland, United States of America; 2 BSP, Science Applications International Corporation-Frederick, Inc., National Cancer Institute-Frederick, Frederick, Maryland, United States of America; 3 Bacteriology Division, United States Army Medical Research Institute of Infectious Diseases, Frederick, Maryland, United States of America; Instituto Butantan, Brazil

## Abstract

*Yersinia pestis* is the etiologic agent of plague that has killed more than 200 million people throughout the recorded history of mankind. Antibiotics may provide little immediate relief to patients who have a high bacteremia or to patients infected with an antibiotic resistant strain of plague. Two virulent factors of *Y. pestis* are the capsid F1 protein and the low-calcium response (Lcr) V-protein or V-antigen that have been proven to be the targets for both active and passive immunization. There are mouse monoclonal antibodies (mAbs) against the F1- and V-antigens that can passively protect mice in a murine model of plague; however, there are no anti-*Yersinia pestis* monoclonal antibodies available for prophylactic or therapeutic treatment in humans. We identified one anti-F1-specific human mAb (m252) and two anti-V-specific human mAb (m253, m254) by panning a naïve phage-displayed Fab library against the F1- and V-antigens. The Fabs were converted to IgG1s and their binding and protective activities were evaluated. M252 bound weakly to peptides located at the F1 N-terminus where a protective mouse anti-F1 mAb also binds. M253 bound strongly to a V-antigen peptide indicating a linear epitope; m254 did not bind to any peptide from a panel of 53 peptides suggesting that its epitope may be conformational. M252 showed better protection than m253 and m254 against a *Y, pestis* challenge in a plague mouse model. A synergistic effect was observed when the three antibodies were combined. Incomplete to complete protection was achieved when m252 was given at different times post-challenge. These antibodies can be further studied to determine their potential as therapeutics or prophylactics in *Y. pestis* infection in humans.

## Introduction

*Yersinia pestis* (*Y. pestis*) is the causative agent of plague that has killed over an estimated 200 million people in previous pandemics [1]. The current incidence of plague is low but the animal reservoirs for *Y. pestis* exist worldwide. Sporadic cases have been reported recently with an average case number of 2,500 worldwide [2]. *Y. pestis* can be rendered airborne and its potential use as a bioweapon is recognized [3] as a category A agent on the NIAID list of biodefense-related pathogens. Current treatment for plague consists of antibiotics, while a live attenuated vaccine against plague is used in the former Soviet Union for prevention [4]. Nevertheless, these live attenuated whole-cell vaccines or killed whole-cell vaccines have adverse effects to varying degrees [4]. Though both types of treatment are efficacious, there is a need for an alternative treatment for plague [5].

A multiple-antibiotic-resistant isolate of *Y*. *pestis* has been isolated, and drug resistance was shown to be mediated by a self-transferable plasmid [6], [7]. A subunit vaccine, which consists of two virulent factors, the F1 protein and V-antigen, is currently in human clinical trials [8]–[10]. Studies involving the vaccine antigens in various formats have provided the proof-of-concept data that humoral response can be efficient in protection against *Y. pestis* [11], [12]. There are multiple reports that mouse anti-plague monoclonal antibodies (mAbs) against a *Y. pestis* challenge can passively protect a mouse against plague [13]–[15]. Therefore, mAb therapy may be an attractive alternative to the existing treatments for plague. Despite the promising possibilities, there remains a major hurdle in the treatment against plague and that is the possible immune response of humans to the mouse mAbs that are currently available. One possibility to ameliorate the immune response against the mouse mAb is to humanize the mAb for use in humans, or another alternative is to develop new and fully human anti-plague monoclonal antibodies for clinical usage [16].

We describe here the isolation of three mAbs from a large naive human phage-displayed Fab library. One, designated as m252, is against the F1- antigen and the other two (m253, m254) are against the V-antigen. When used alone, m252 displayed good protective effects, whereas m253 and m254 did not. However, a clear synergistic effect was found when they were used together. Maximum protection by m252 alone could be achieved by altering the antibody administration schedule. This is the first report describing the isolation of fully human anti-plague mAbs that show efficacy in a mouse model of plague. These antibodies represent a significant breakthrough toward possible adjunctive therapeutic treatment of *Y. pestis* infection in humans.

## Results

### Selection and purification of human anti-F1 and anti-V Fabs

**Figure 1:**
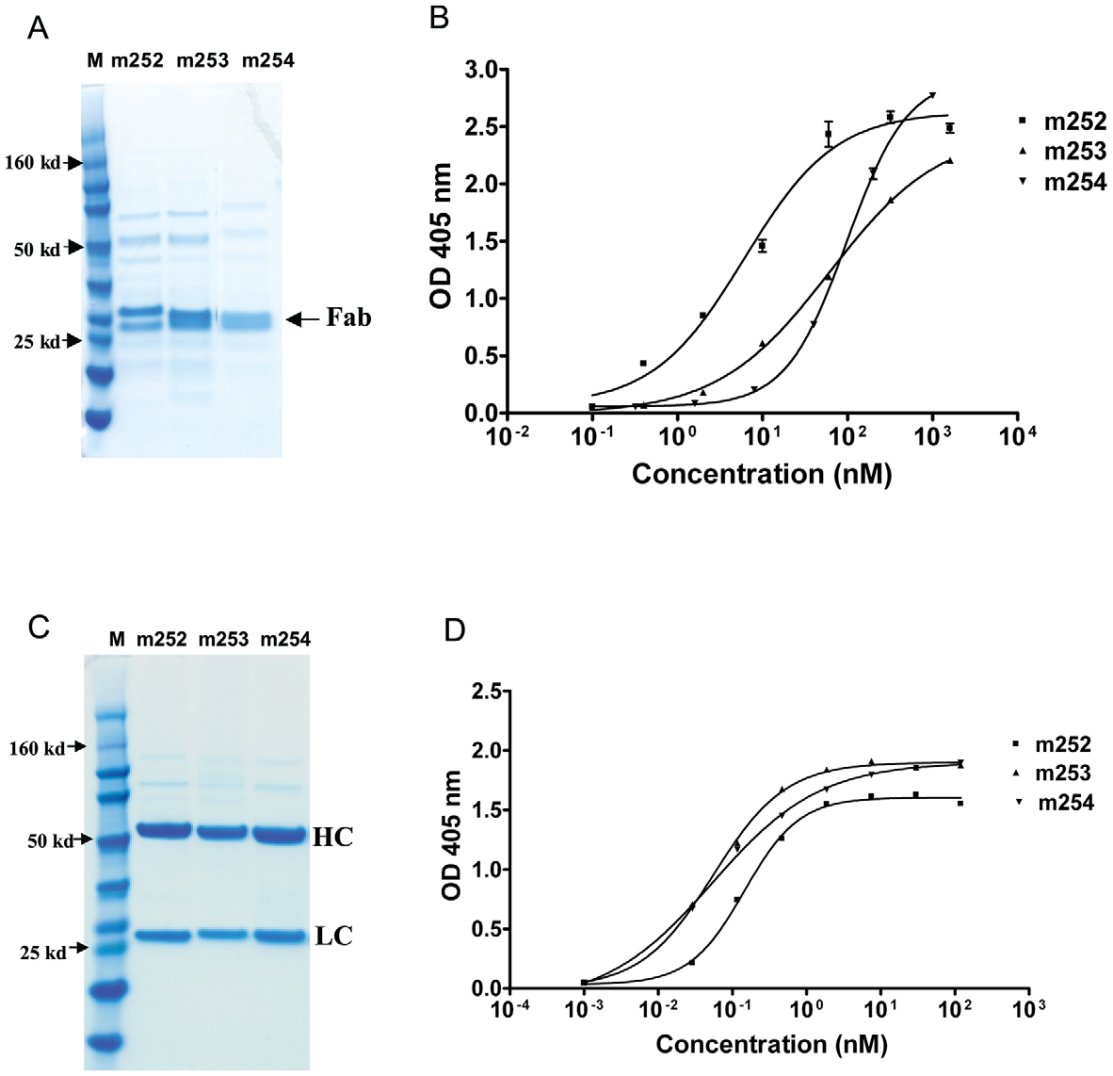
Characterization of the anti-F1 and anti-V antigen human antibodies. A. Anti-F1 human antibody m252 and anti-V human antibodies m253 and m254 were expressed as Fabs, purified, and analyzed on a reducing SDS-PAGE gel. B. Specific binding by the Fabs antibody fragments to their respective antigens in an ELISA assay. C. The three human anti-F1 and anti-V antibodies were expressed as IgG1s, purified and analyzed on a reducing SDS-PAGE gel. D. Specific binding of the purified IgG1s to their respective antigens in an ELISA assay.

With the F1 antigen, only the plate format yielded positive Fab clones after four rounds of selection with the F1 antigen. Sequencing of the clones confirmed that they were identical and designated as m252. With the V-antigen, the plate and bead format each yielded two positive Fab clones after four rounds of selection with each format. One clone from each format, designated as m253 and m254, respectively, was selected for further analysis. Sequence analysis revealed that m252 has heavy and light chains originated from germlines IGHV1-2*02 and IGKV1-16*01 respectively. M253 originated from IGHV1-18*01 and IGKV1-9*01, while m254 was from IGHV3-43*01 and IGKV1-27*01. The mutational rate ranged from zero to less than 10%. This is typical for antibodies isolated from naïve human libraries by panning against viruses causing acute infection in contrast to neutralizing antibodies selected from immune human libraries by panning against HIV-1, which causes a chronic infection [17]. Each of the clones was then transformed into HB2152 cells and the respective Fab was expressed and purified (Figure 1a). After conversion to IgG1 expressing clones, the three antibody clones were transiently transfected into Freestyle HEK 293F cells, and the expressed IgG1s were purified (Figure 1c).

### Binding of the selected mAbs as Fabs and IgG1s to their antigens

To determine both the specificity and affinity of the selected antibodies, ELISA with both Fab and IgG formats were conducted as described in the methods. All Fabs and IgGs bound to their respective antigens specifically without cross-reaction to other antigens tested (Figure 1b and d). Anti-F1 Fab and IgG have apparent affinities in the low and sub-nM range, respectively. Both m253 and m254 Fabs have apparent affinities of approximately 100 nM (Figure 1b and d). Their IgGs however have sub-nM apparent affinities (avidities). The avidity effect is very pronounced for all three antibodies.

### Low level of competition between the human anti-F1 and anti-V Fabs and mouse anti-F1 and anti-V mAbs

**Figure 2:**
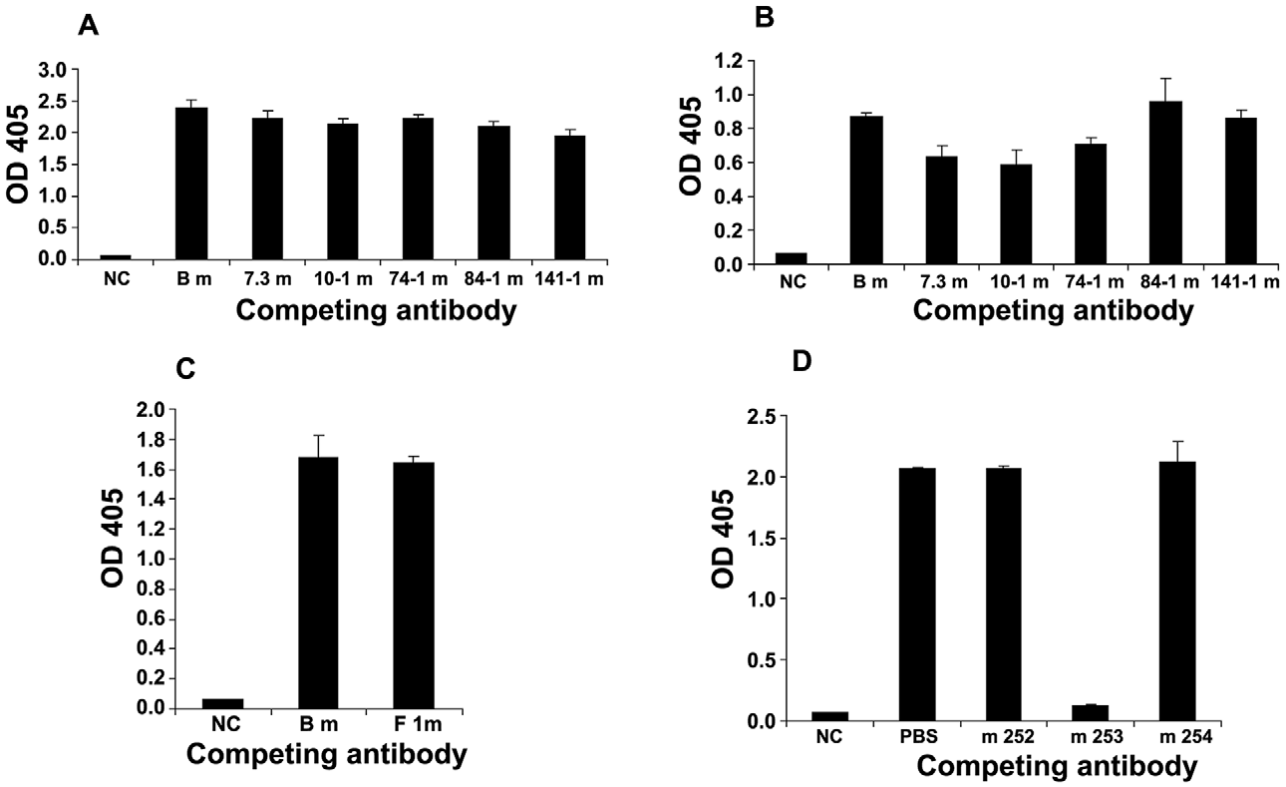
Epitope binding analysis of human Fab by competition-ELISA. A and B, Fab anti-V m253 and m254 binding to the V-antigen were analysed by competitions with equal amount of control IgG1, mouse anti-*Burkholderia mallei* (Bm), and five other mouse anti-V mAbs (7.3 m, 10–1 m, 74–1 m, 84–1 m, 141–1 m) as described in Materials and Methods, respectively. The amount of bound human Fab was then determined. C. Lack of competition between human Fab anti-F1 fragment and mouse anti-F1(F1m) mAb. D. Competition among human antibodies. Fab m252, m253, and m254 were mixed with equal amounts of IgG m253 and applied to an ELISA plate coated with their respective antigens. The amounts of bound Fab were determined. PBS indicates m253 mixed with an equal volume of PBS buffer only. In all panels, NC indicates samples with no primary antibodies added serving as secondary antibody controls.

The three human anti-plague Fabs were used in competition-ELISAs against a panel of mouse anti-plague mAbs. The mouse anti-plague mAbs included the anti-F1 mAb F1-04-A-G1, and five anti-V mAbs, which included the anti-V mAb 7.3 m that was highly protective. We found no apparent competition between the human anti-V m253 Fab and the mouse anti-V mAbs (Figure 2a). However, we observed some weak competition between the human anti-V m254 Fab antibody and some of the mouse anti-V mAbs (7.3 m, 10–1 m, and 74–1 m) that we did not see with the human anti-V m253 Fab antibody (Figure 2b). The competition between the human anti-F1 m252 Fab antibody and the mouse anti-F1 mAb was also minimal (Figure 2c). We ran two controls in the competition studies with the human and mouse mAbs. For one control we did not add a primary antibody (labeled NC, Figure 2a–2c), and for a second control we used a nonspecific mouse isotype IgG1 mAb in the competition assay (labeled Bm, for a *Burkholderia mallei* IgG1 mAb). There was also a lack of competition between the human anti-V m253 and m254 Fab antibodies, suggesting that these two human anti-V Fabs recognize different epitopes (one which may be conformational) on the V-antigen (Figure 2d). Of note, however, is that when the competition-ELISA was performed in a different fashion, namely when the human anti-F1 or anti-V mAbs were allowed to bind to the respective antigens before adding the mouse anti-F1 or anti-V mAbs, moderate competition was detected between the human anti-F1 m252 and the mouse anti-F1 mAbs, as well as between the human anti-V m253 and mouse anti-V 84-1 mAbs (data not shown).

### Epitope mapping by peptide-ELISA

**Figure 3:**
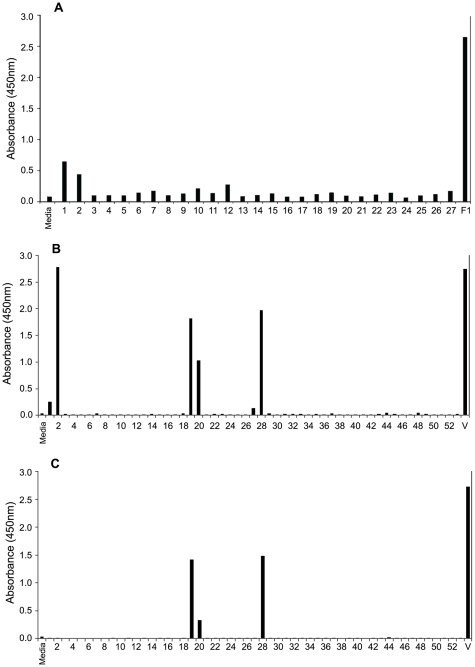
Epitope mapping of the human anti-F1 and anti-V antibodies by peptide binding assay. A. Each of twenty-seven peptides that covered the full length of the F1-antigen were used to coat an ELISA plate (0.05 ml of a 25 µg/ml solution of each peptide), and binding by the human anti-F1 m252 mAb (0.05 ml of a 10 µg/ml solution) was analyzed. The sample labeled F1 was the full-length antigen used to coat the plate as the positive control (0.05 ml of a 2 µg/ml solution), and the sample labeled Media was the negative control with no primary antibody added. B and C. Each of fifty-two peptides that covered the full length of the V- antigen was used to coat an ELISA plate (0.05 ml of a 25 µg/ml solution), and the human anti-V m253 (B) and m254 (C) mAbs were used (0.05 ml of a 10 µg/ml solution), respectively, to analyze for binding. The sample labeled V was the positive control (0.05 ml of a 2 µg/ml solution), and the sample labeled Media was the negative control without the primary antibody.

To characterize the binding of the human anti-F1 and anti-V mAbs to the F1- and V-antigens, respectively, more closely, we examined the binding of the human mAbs to two separate panels of overlapping peptides. One panel covered the full-length of the F1 antigen (27 peptides) and the other - the V antigen (53 peptides). For the human anti-F1 m252 mAb, there was a weak-moderate binding signal with peptides 1 and 2, which are located at the N-terminus of the F1-antigen (Figure 3a). This suggests that the m252 mAb may also recognize a conformational region that involves peptides 1 and 2. The binding by human anti-V m253 mAb resulted in a strong signal with peptide 2 and a weak signal with peptide 1 (Figure 3b). The anti-V m254 mAb, did not bind to any of the peptides, suggesting that its epitope may be conformational (Figure 3c). In initial studies with m254 we saw some weak binding to peptides 36 and 42, but when we repeated the binding studies with the human anti-V mAbs we did not see binding to peptides 36 and 42. This might explain its weak competition with mouse antibodies that recognize diverse epitopes on the V-antigen (Figure 2b). The positive signals seen with the m253 and m254 mAbs with V-antigen peptides (numbers 19, 20, 27, and 28) are nonspecific signals that are generated by the secondary antibody (Amemiya et al. unpublished). The epitopes of the mouse antibodies have also been determined by the peptide binding assay (Amemiya, et al. unpublished). The data is consistent with the competition-ELISA presented in this study.

### Specific binding of human anti-F1 IgG1 to *Y. pestis*

**Figure 4:**
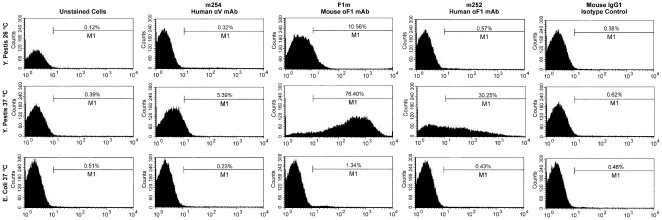
Specific binding of the human anti-F1 IgG1 to the *Y. pestis* cell surface was detected by a flow cytometry assay. A mouse anti-*B mallei* IgG1was used (10 µg/ml) as an isotype mAb control, and the mouse anti-F1 (F1-04-A-G1, F1m) mAbs was used (10 µg/ml) as a positive IgG1 control. *Y*. *pestis* grown at 26°C (top panel) was used as an anti-F1 mAb negative control. *E.coli* grown at 37°C was used as a specificity control for anti-F1 and anti-V IgGs.

**Figure 5:**
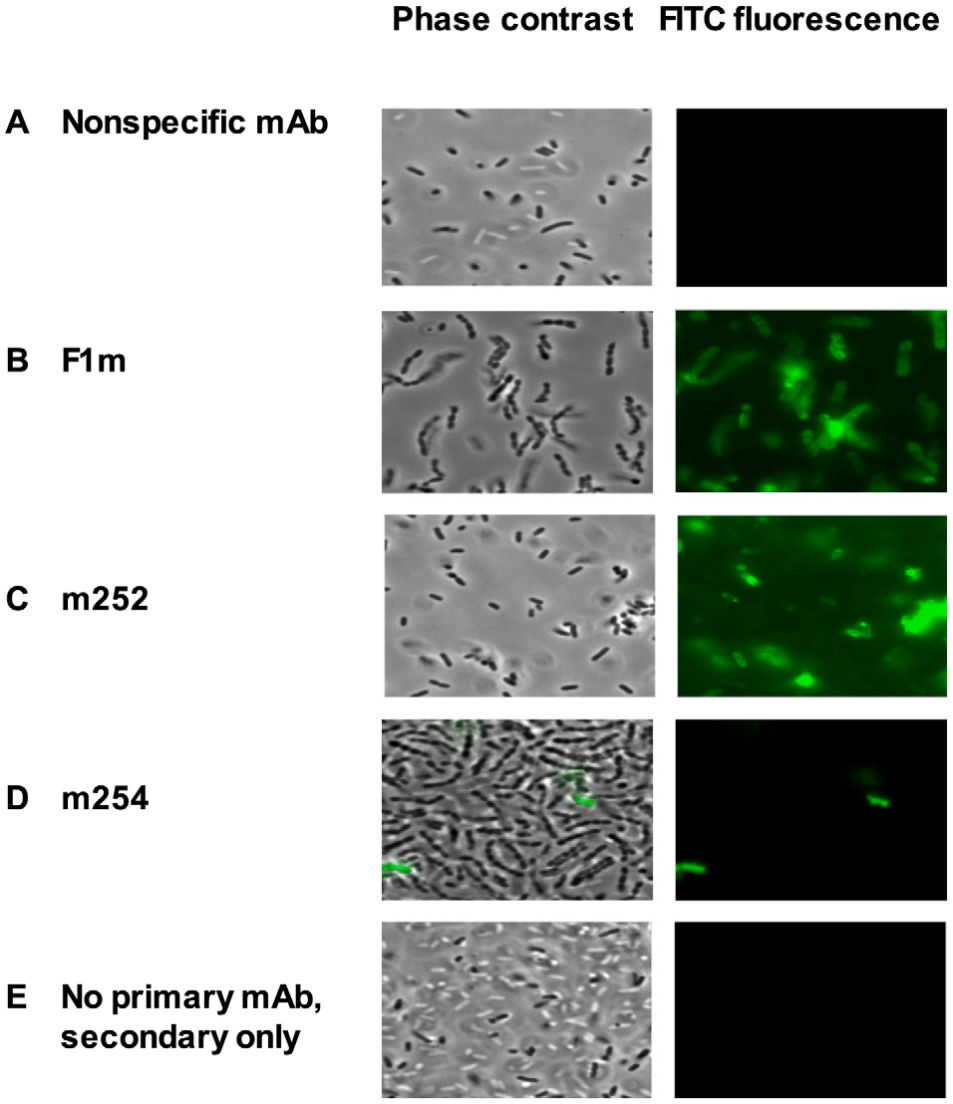
Immunofluorescence detection of human anti-F1 IgG1 mAb (m252) binding to the *Y*. *pestis* cell surface. The mouse anti-*B mallei* IgG1 (panel A) was used as a nonspecific isotype control. The mouse anti-F1 mAb (F1m) (panel B) was used as a positive control. The binding by the human anti-F1 mAb (m252) is shown in panel C. The rare binding shown with the human anti-V m254 to *Y. pestis* (panel D) was considered to be the result of autofluoresence because it was seen in both phase contrast and fluorescent microscopy. In the bottom panel E, there was no primary antibody in the reaction before the secondary antibody was added as a negative control.

To test if the human anti-F1 and anti-V IgGs bind their respective targets on bacterial cells, we performed flow cytometry analysis. Both the mouse anti-F1 F1m mAb and human anti-F1 m252 mAb bound specifically to *Y. pestis* grown at 37°C and not to the same strain grown at 26°C. Neither did they bind to a control *E. coli* strain grown at 37°C (Figure 4, bottom panel). This is consistent with previous reports that the expression of the F1-antigen is regulated by temperature (37°C), and it is not expressed at RT. The human anti-V m254 mAb showed minor binding to the *Y. pestis* grown at 37°C, although we do not normally see binding with the human anti-V m253 or mouse anti-V mAbs, which included 7.3 m, 74–1 m, and 141–1 m (Amemiya, unpublished). A mouse IgG1 isotype control mAb, which did not show any binding to the whole-cells, was included to show binding by the mouse and human anti-F1 mAbs was antibody specific. To further confirm the binding data we used immunofluorescence technique. The results were consistent with the flow cytometry data, where only the mouse and human anti-F1 mAb bound to *Y. pestis* whole-cells (Figure 5). Neither the human anti-V m254 mAb (Figure 5D) nor mouse anti-V mAbs, like 7.3 m (data not shown) nor control samples (Figure 5A, nonspecific mouse IgG1 isotype; Figure 5E, no primary mAb) showed significant binding to *Y. pestis*.

### Human anti-F1 and V mAbs protect synergistically against a *Y. pestis* challenge in a bubonic plague model

**Figure 6:**
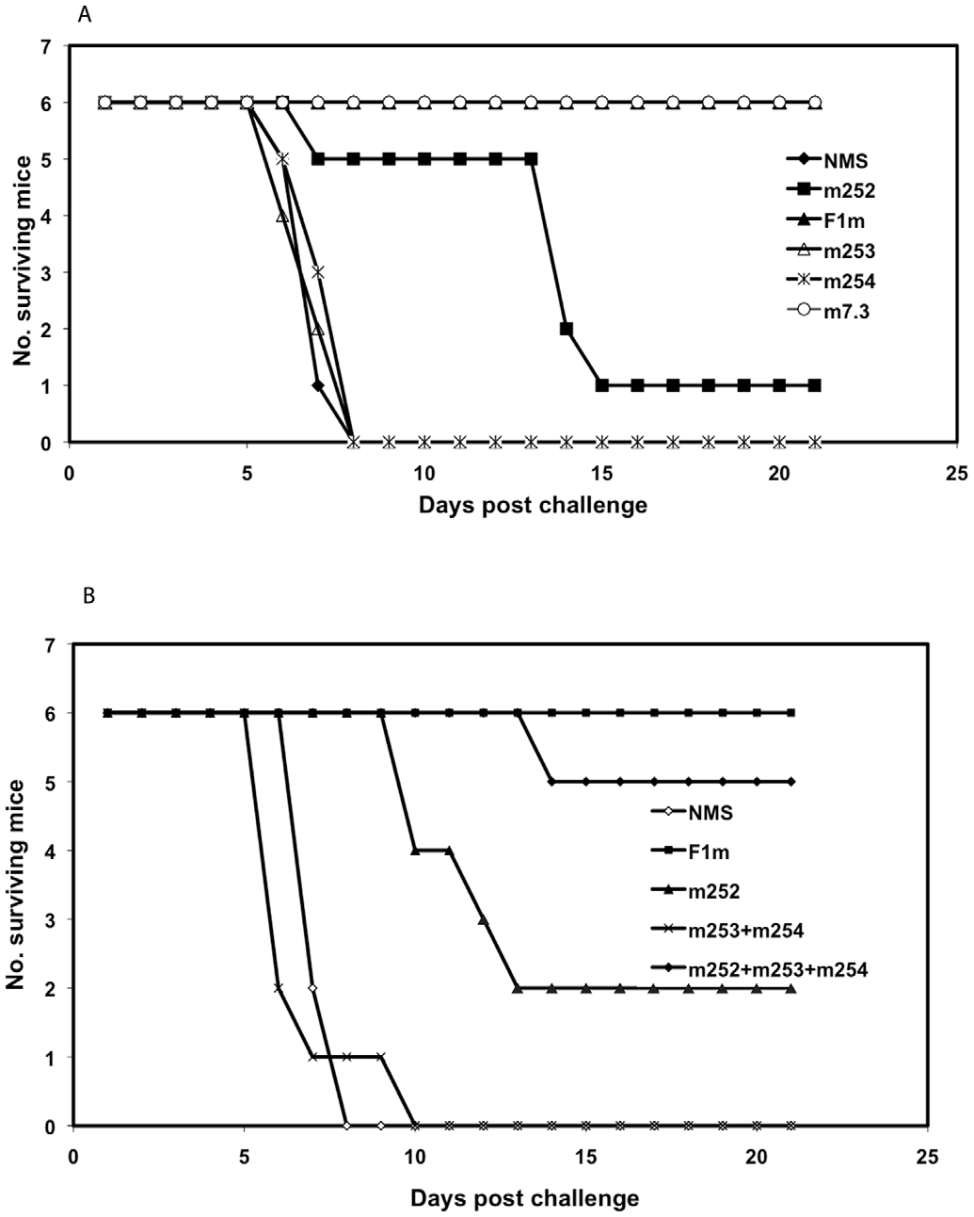
Human anti-F1and anti-V mAbs show synergistic protection when used together in the murine bubonic plague model. The human and mouse anti-F1 and anti-V mAbs were given i.p. to mice 24 hrs before parenteral challenge with *Y. pestis* CO92, and the number of surviving mice for each treatment group was monitored for 21 days after challenge. 6A. The following antibodies and amounts were used: normal mouse serum (NMS, 500 µg); mouse anti-F1 (F1m, 500 µg); mouse anti-V (7.3 m, 100 µg); human anti-F1 (m252, 500 µg); human anti-V (m253 and m254, 500 µg each). 6B. The following mAbs and amounts were used: NMS, 500 µg; F1m, 500 µg; m252, 500 µg; m253+ m254, 500 µg each; m252+ m253+ m254, 500 µg each.

The ability of the human anti-F1 and anti–V mAbs to passively protect mice against a *Y. pestis* infection was evaluated in a bubonic plague model. The human anti-F1 and anti-V mAbs were used either separately or together in different combinations. When mAbs m252 and m253 and m254 were given to mice separately before challenge with *Y. pestis* CO92, only the human anti-F1(m252) mAb showed some efficacy. The mean-time-to-death (MTD) in the m252 mAb-treated mice was shifted to 13.0 days (1/6 survivors) when compared with mice given normal mouse serum (NMS), which had a MTD of 7.0 days (0/6 survivors) (Figure 6A). Unlike m252, however, the human anti-V mAbs, m253 and m254, did not show any significant protection [mean-time-to-death (MTD) of 6.7 days (0/6 survivors) and 7.3 days (0/6 survivors), respectively] when compared to the NMS -treated mice. The mouse anti-F1 (F1m) and anti-V (7.3 m) control mAbs both passively protected all (6/6) mice under the same challenge conditions (MTD of 21 days). When both human anti-V mAbs were given to mice passively (Figure 6B), no improvement in protection after challenge was observed (MTD of 6.8 days, 0/6 survivors), which was similar to that seen with the NMS-treated mice (MTD of 7.3 days, 0/6 survivors). However, when the human anti-F1 m252 mAb (MTD of 11.3 days, 2/6 survivors) was given together with the two human anti- V m253 and m254 mAbs, a greater number of mice were passively protected (MTD 14.0, 5/6 survivors) than when the antibodies were used separately, suggesting a synergistic effect.

**Figure 7:**
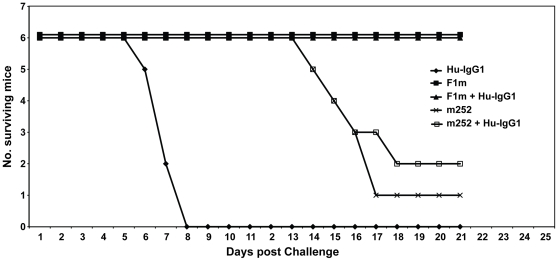
A nonspecific human IgG1 antibody (Hu-IgG1) had little effect on the mean-time-to-death (MTD) and number of surviving mice that received the human anti-F1 mAb. The nonspecific human IgG1mAb, and the human and mouse anti-F1 and anti-V mAbs were given i.p. to mice 24 hrs before parenteral challenge with *Y. pestis* CO92, and the number of surviving mice for each treatment group was monitored for 21 days after challenge. Mouse or human mAbs and their amounts were given to the following groups of mice: nonspecific human IgG1 (Hu-IgG1, 1,500 µg); F1m (F1m, 500 µg); F1m (500 µg) + Hu-IgG1(1,000 µg); m252, 500 µg; m252 (500 µg) + Hu-IgG1 (1,000 µg).

Although we saw some protection with the human m252 mAb, and a synergistic protective effect when the human m252 mAbs was given with the human m253 and m254 mAbs in the bubonic plague model, we wondered if there was any effect (positive or negative) with a nonspecific human IgG1 mAb by itself or when combined with the human anti-F1 m252 mAb in the number of survivors in the mouse model of plague. To answer this question, we injected five groups of mice with the following mAbs: one group of mice with only a nonspecific human IgG1 mAb (Hu-IgG1, 1500* µg*); another group of mice with the mouse anti-F1 mAb (F1m, 500* µg*); another group of mice with the human anti-F1 mAb (m252, 500* µg*). Two other groups of mice were included, that were given either F1m (500 µg) or m252 (500 µg), with the nonspecific Hu-IgG1 mAb (1000* µg*) one day before challenge with *Y. pestis* CO92 (Figure 7). All mice in the group that received only the nonspecific Hu-IgG1 mAb died by day 8, which was similar to the control antibody mouse groups in Figure 6A and 6B. The same number of survivors was obtained (6/6) whether mice were given only mouse F1m mAb or F1m combined with the nonspecific Hu-IgG1. As we have seen previously (Figure 6A), only 1/6 mice survived in the group that received only human m252 mAb. When the human m252 mAb was combined with the nonspecific Hu-IgG1 mAb, we obtained one more survivior (2/6) than we obtained without the nonspecific Hu-IgG1 mAb. This variation in the number of survivors was not different than we saw previously (Figure 6B). In addition, the MTD was not affected by the presence of the nonspecific Hu-IgG1 (15.8 days) when given with the Hu-antiF1 mAb (15.8 days). These results suggest that the nonspecific Hu-IgG1 mAb had little effect on the survival of mice given the human m252 mAb or mouse F1m mAb.

### Delaying time of delivery of human anti-plague mAbs provided better protection against a plague challenge

**Figure 8:**
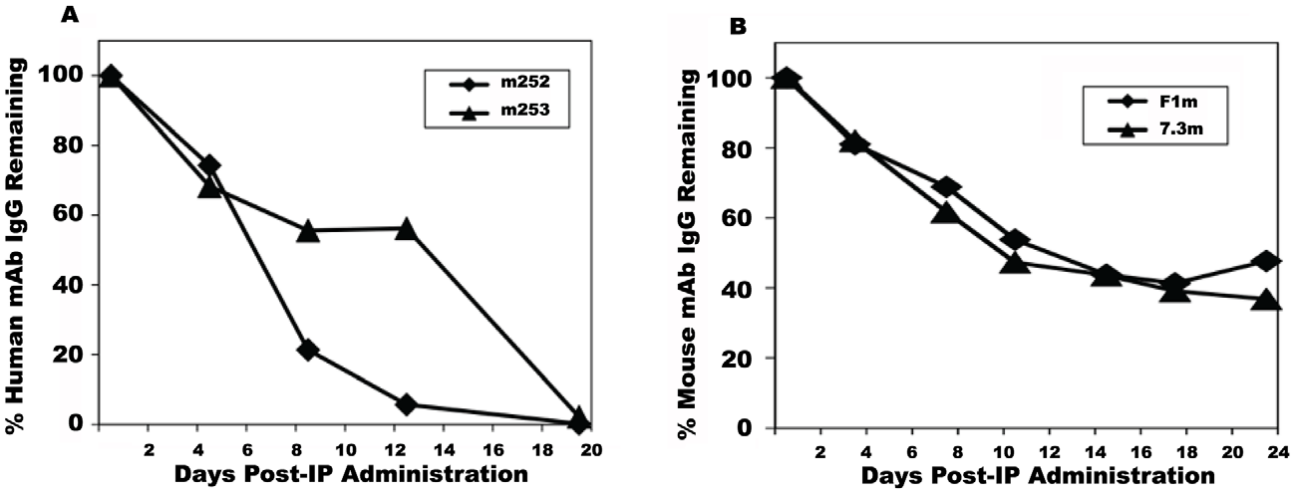
Serum concentrations of human and mouse anti-F1 and anti-V mAbs in the plague mouse model over time. The same amount of human IgGs (500 µg) as used in the challenge studies were administered via i.p. and blood samples were collected at indicated time points. The human IgG concentration was determined by ELISA. The data shown are averages from three mice for each IgG. A. The amount of remaining human anti-F1 m252 and anti-V m254 in mouse serum. B. The amount of remaining mouse anti-F1 F1m and anti-V 7.3m in mouse serum.

One possible reason we observed less protection with the human anti-F1 and anti-V mAbs in the mouse plague model was that the level of the human IgGs may not have been sustained in the mouse over time compared to the mouse IgG mAbs, We tested this hypothesis in two separate studies. We first examined the concentration of the human antibody in mice directly, by measuring the level of m252 (anti-F1) and m253 (anti-V) in serum after they were given the human mAbs by i.p. injection. Mouse sera were collected at different time points after the initial dosing and human IgG levels were monitored by direct ELISA. As seen in Figure 8A, the anti-F1 m252 mAb appeared to have a half-life of approximately 8 days, and the half-life of m253 mAb was approximately 10 days. After 21 days, the levels of these two human antibodies were undetectable. In contrast, the level of both the mouse anti-F1 (F1m) and anti-V (7.3 m) mAbs may have decreased initially like the human anti-plague mAbs, but after 21 days, the levels were still approximately 40–50% of the initial concentration (Figure 8B). The half-live of human IgG mAbs in mice reported here is similar to what was found in another study where human mAbs were used against another biothreat agent [18]. In contrast, a human IgG molecule would have an average serum half -life of 21 days in a human [19].

**Figure 9:**
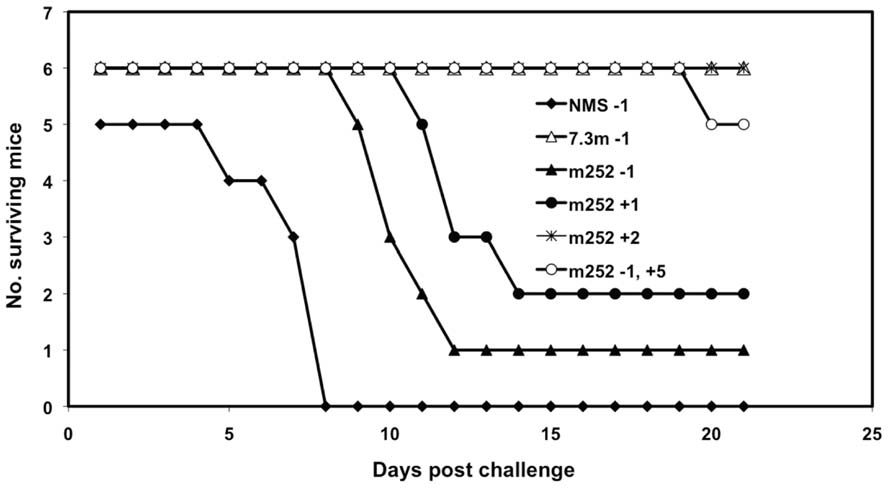
Post-challenge administration of the human anti-F1 m252 mAb conferred better protection. The human anti-F1 m252 mAb was administered before or after *Y*. *pestis* challenge, and mice were monitored for 21 days after challenge. The mouse anti-V 7.3m mAb (100 µg) was used as a positive control mAb. Normal human serum (NHS, 500 µg), which was used as a negative control, had only 5 mice per group. The numbers behind each antibody represent the time in days in which the antibody was administered (500 µg) to mice relative to the day of challenge (day 0). The two numbers after the human anti-F1 m252 (−1 and +5) represent two different days when the mAb (500 µg) was added to the same group of mice relative to the day of challenge.

Because of these findings we then administered the anti-F1 m252 mAb at different time points relative to the time of challenge. While the original regimen provided consistently modest protection, administration of the human m252 mAb 24 and 48 hours post-challenge provided increasing protection with the 48 hours schedule provided complete protection (Figure 9). Antibody administration at even later time points was not performed since mice began to die 3–4 days after challenge without any treatment. However, we did evaluate the effect of a second dose of antibody at a later time point. In this group, mice first received an initial dose of human anti-F1 m252 mAb 24 hour before challenge as was done with the earlier protocols. These mice then received a second dose of the human anti-F1 m252 mAb 5 days after challenge. There was an increase in both the number of survivors (5/6) and MTD (20 days) approaching the efficacy displayed by a single dose administered 48 hour after challenge. These data suggest that the optimum serum concentration of the human IgG1s was critically dependent on the time of administration, and that the optimum concentration of the human anti-plague IgG1s in turn determined the outcome of the treatment protocol.

## Discussion

Antibiotics have been at the forefront of combating bacterial infection for decades with great success. However, the development of new antibiotics is struggling to keep pace with the emergence of drug resistant bacterial strains, for example as in *Y. pestis* [6]. There has been an intense interest in developing antibody-based therapies as an alternative method of treatment [5]. Initially, antibody-based therapy was mostly limited to treating cancer or immune disorders. However, because of a better understanding of the pathogenesis of infectious agents, and the advancement in the development of protective or neutralizing antibodies, the use of antibody-based therapy against infectious agents has become more frequent [20]. In this report we described the first isolation of fully human mAbs against the *Y. pestis* virulence factors F1- and V-antigens. Previous studies have shown that mouse mAbs can be effective in protecting mice against *Y. pestis* ([13]–[15]. However, because they are mouse mAbs they are not safe to use in their present form in humans [21]. Of particular concern is the immune reaction against mouse primary antibody sequences in human system. This may lead to severe adverse effects and at the same time reduce the potential benefits. It is highly desirable to have fully human antibodies for these reasons. The fully human anti-F1 (m252) reported here displayed moderate to good protection against a bubonic plague challenge with *Y. pestis* CO92. On the other hand, the two anti-V mAbs (m253, m254) when used separately did not show any efficacy, but when they were used together with m252, the combination of the human anti-plague mAbs resulted in better protection overall, suggesting a synergistic effect between the antibodies. A similar effect was reported in studies using mouse anti-F1 and anti-V mAbs in a mouse model of plague [15]. Further in our case with the human anti-F1 mAbs, when we gave mice the human anti-F1 mAb 1–2 days after challenge, we saw a greater protection against a plague challenge could be achieved. This suggested that the maintenance of serum concentration of the human mAbs in the mouse was possibly one critical factor for better protection. Kinetic studies revealed that indeed the serum concentration of the human antibodies dropped further than the mouse anti-plague mAbs over the course of the study. It is also plausible that the human anti-plague mAbs might bind to other mouse antigens nonspecifically, thus decreasing the amount of free circulating human anti-plague antibody in the mouse.

Another important underlying factor for efficient protection by antibodies is the epitopes the antibodies recognize. Although the human anti-F1 (m252) mAb appeared to bind to the same region as the mouse antiF1, which was at the amino-terminal end of the F1-antigen (Amemiya et al., unpublished), we could not demonstrate direct competition between these two antibody species. This observation may be the result of the nature of the antibody binding site or epitope, because several mouse mAbs isolated independently recognized the same region or epitope on the F1-antigen, behaved in the same manner (Amemiya et al., unpublished). It may be that once these mAbs bound to the amino-terminal end of the F1-antigen, they may not readily come off the protein or may dissociate very slowly. Whether this is because the binding site involved both linear and conformational sites is not known, but both the mouse and human anti-F1 mAbs bound to the whole anti-F1 antigen very well, but only weakly – moderately to the 5′-peptides. Nevertheless, the human m252 mAb was as protective as the mouse anti-F1 mAb when the human mAb was given after challenge. It has also been reported that a neutralizing epitope on the V-antigen was located in a region spanning amino acids 135 to 275, and a possible minor, secondary neutralizing epitope exists near the amino-terminal region of the V-antigen [14]. Neither of our human anti-V antibodies reported here competed with the mouse anti-V antibodies efficiently. The minor competition between the human anti-V Fab antibody and the mouse anti-V antibodies suggests that the recognition site of the human anti-V antibodies is slightly different or they may partially share conformational binding site. These differences might be one reason for their inability to protect as efficiently as the human anti-F1 m252 mAb.

Exactly how the human anti-F1 252 m mAb is able to protect mice may be directly related to the presence of F1 antigen on the surface of the plague organism. The F1-antigen has been reported to be anti-phagocytic [22], [23]. The ability of macrophages to take up the plague organism is directly related to the lack of the F1-antigen, and resistance to phagocytosis is related to the presence of the F1-antigen. The binding of the human anti-F1 252 m mAb to the surface of the plague bacilli or opsonization may trigger phagocytosis of encapsulated bacilli into macrophages, thereby allowing phagocytic cells to clear the host of the pathogen.

The exact mechanism by how the V-antigen exerts it virulence is not completely known. There are reports showing that V-antigen is secreted into the growth medium and the secretion is important for virulence [24], [25]. The secretion of V-antigen in the medium has been described to be dependent on contact with the host cell, and could also be directed into the host cell by a *Yersinia* outer proteins (Yops) dependent secretion (Ysc) type III system (TTSS) [26]–[28]. It also has been suggested that free V-antigen may enter the cell by endocytosis besides being injected into the cell by the Ysc TTSS [29]. Once inside the host-cell, we do not know exactly what host proteins interact with the intracellular V-antigen [29]. Nevertheless, there is some evidence that anti-V antibodies enhance phagocytosis through possibly the Fc receptor, and thereby block Yop delivery into the host cell, and thus preventing Ysc dependent TTSS injection of V-antigen into the host cell [30], [31].

In this study, however, we were not able to detect binding of the human anti-V mAbs on the surface of *Y. pestis* cells by flow cytometry or fluorescent microscopy, suggesting that the V-antigen was not on the bacterial cell surface under the conditions used in our studies or the expression level of V-antigen was below the level of detection. As has been discussed previously, however, the presence of V-antigen on the surface of the cell may be dependent on contact with the eukaryotic host cell. The highly specific region of the neutralizing epitope (s) on the V-antigen suggests a possible ligand-receptor interaction between the V-antigen and a cellular factor. This indicates that perhaps the V-antigen exerts its biological effect through mechanisms other than mediating the TTSS pathway.

In conclusion, the human anti-plague antibodies reported here represent perhaps the ones that are closest to practical clinical use. They may be safer and more efficient in the human system due to their fully human nature, and a likely longer half-life in humans. Also, intravenous application of the antibodies in humans may be a rapid delivery system that may augment antibiotics treatment in plague-exposed individuals. Finally, the affinity of all three antibodies can be further increased using readily available techniques, may reduce the dose required for efficient protection. The successful development of these three human anti-plague antibodies in this model suggests that new and more potent anti-V antibodies can be potentially developed using the same approach but with restricted V-antigen subunit fragments containing the critical neutralizing epitopes, and perhaps other virulent factors.

## Materials and Methods

### Bacterial strains and cultivation

The *Y. pestis* CO92 strain used in the challenge studies was originally obtained from T. Quan, Centers for Disease Control and Prevention, Fort Collins, Co. It was isolated from the sputum of a human case of pneumonic plague [32]. The *Y. pestis* CO92 was grown and inoculum prepared for challenges essentially as described previously [33]. A *Y. pestis pgm-* strain, which was originally isolated from *Y. pestis* CO92, and used in the antibody binding studies described below was obtained from Susan L. Welkos (USAMRIID, Frederick, MD).

### Proteins and peptides

*Y. pestis* purified F1,V, and F1-V [34] protein antigens were obtained from Brad Powell (USAMRIID, Fort Detrick, Frederick, MD). The 27-peptide array that covered the F1-antigen were 14- to 17-mers with 11 amino acid overlaps; they were obtained from the Biodefense and Emerging Infections Research Resources Repository (BEI)(Manassas, VA). The 53-peptide array that covered the V-antigen were 15- to 17-mers with 11 or 12 amino acid overlaps and were obtained from BEI.

### Mouse and human monoclonal antibodies

The anti-F1 mouse mAb F1-04-A-G1 (or mF1) was provided by George Anderson (USAMRIID) [13], and anti-V mouse mAbs 10–1 m, 74–1 m, 84–1 m, and 141–1 m as well as the control IgG1 mouse anti-*Burkholderia mallei* (Bm) antibody were obtained from Sylvia Trevino (USAMRIID) and anti-V mouse mAb 7.3 (7.3 m) was obtained from Jim Hill (Porton Down, Wiltshire, UK) [14] and used in competition ELISAs and as positive controls in mouse passive protection experiments. All mice mAbs were IgG1 isotypes. The human IgG1 control mAb used in the passive protection study was an anti-human IGFII mAb.

### Selection of anti-F1 and V Fabs

Purified F1-and V-proteins were either coated directly to Maxisorp plates (Nunc, Denmark) in PBS buffer at 4°C, overnight for plate format panning or were biotin-labeled first with EZ-link Sulfo-NHS-LC-Biotin (Pierce, Rockford, IL) for streptavidin-conjugated magnetic bead format panning. The labeling was performed according to the manufacture's recommended protocol. For the plate format, approximately 10^12^ Fabs displayed on the surface of phage amplified from a large naive library [35] were suspended in PBS with 2% dry milk and applied to wells coated with the F1- or V- proteins. After incubating for 2 hours at room temperature, each well was washed 5 times for the first round and 10 times for the subsequent four rounds before the phage were rescued with TG1 cells at the exponential growth phase. For the bead format, biotin-labeled F1- and V-antigens were first incubated with the same amount of phage as in the plate format in 1 ml of PBS+2% dry milk suspension at room temperature for 2 hour. Fifteen µl of Dynabeads MyOne Streptavidin T1(Invitrogen Dynal AS, Oslo, Norway) pre-blocked with PBS+2% dry milk was then added to the antigen/phage mixture for one hour at room temperature. The beads were then washed 5 times with PBS for the first round and 10 times for the subsequent four rounds of selection. Phage were then rescued with TG1 cells. A total of four rounds were performed for each antigen with each format. Monoclonal ELISA was then performed to select for positive clones. One hundred clones were screened for each antigen from each format. Only clones displaying an OD405>2.0 were selected for plasmid preparation and sequencing.

### Expression, purification, conversion to IgG1, and generation of stable clones

Clones selected as described above were transformed into *E.coli* strain HB2151 for expression [36]. Briefly, a single clone was inoculated into 2YT supplemented with 100 units of ampicillin and 0.2% glucose and incubated at 37°C with shaking. When the OD600 reached 0.6–0.9, IPTG was added to achieve a final concentration of 1 mM and the culture was shifted to 30°C with shaking and incubated overnight. Cells were then collected, and lysed with polymyxin B (Sigma, St Louis) in PBS, and mixture subjected to Ni-NTA agarose bead (Qiagen, Hilden, Germany) purification. For IgG1 production, the heavy and light chains of the respective Fabs were cloned into the bi-cistronic expression vector pDR12 kindly provided by Dennis Burton (Scripps Research Institute, La Jolla, CA). For small scale IgG1 production, transient transfection and expression in Freestyle HEK 293F cells (Invitrogen, Carlsbad, CA) were used. For large scale production, stable clones were generated using CHO-K1 (ATCC, Manassas, VA) cells. Briefly, the heavy and light chains of the three human anti-plague IgG1s were cloned into pDR12 vectors and transfected into CHO-K1 cells. One day after transfection, the cells were re-plated and subjected to selection in GMEM medium supplemented with 25 µM MSX. Two weeks later, the MSX resistant clones were amplified further. The clones were tested for the expression of respective IgG1s and then adapted to growth in serum-free medium HyQSFM4CHO (HyClone, Logan, UT) supplemented with 30 µM MSX. The serum-free growth medium was then collected and passed through a protein A-sepharose resin column for IgG1 purification.

### Characterization of the binding by the human anti-F1 and anti-V Fabs and IgGs

An ELISA assay was used to assess the binding ability of the Fabs and IgG1s. Briefly, F1- and V-antigens were coated to a Costar high binding 96-well plate (Corning, Corning, NY) and incubated overnight at 4°C. The next day, the plate was blocked with 2% dry milk in PBS before serial dilution of Fabs or IgGs were applied to the plate. After an incubation of the plate at 37°C for one hour, anti-His-horse radish peroxidase (HRP) for Fab detection or anti-human-Fc-HRP (for IgG detection) in PBS+2% dry milk was added to each plate and incubated for another hour at 37°C. The plates were then washed four times, and the ABTS substrate (Roche, Mannheim, Germany) was added. After approximately 10 min at room temperature, the OD405 was taken.

For competition studies between the mouse mAbs and human Fabs the antigen at 2 µg/ml (F1-protein or V-antigen) was used to coat 96-well plates (Immulon 2HB, Thermo Electron, Milford, MA), and the plates were incubated overnight at 4°C. After washing the plates, a blocking solution (1% bovine serum albumin with 0.05% Tween 20 in PBS) was added to the plates, and plates incubated for 1 hr at 37°C. The flag-labeled human Fabs, and biotinylated-mouse mAb were allowed to bind to the antigen simultaneously for 1 hr at 37°C. To detect the presence of the human Fab, an anti-flag-M2-peroxidase conjugate was used, and to detect the amount of biotinylated mouse mAb present, a streptavidin-conjugated HRP was added for 1 hr at 37°C, before adding a hydrogen peroxidase-3,3′,5,5′-tetramethylbenzidine solution. The color reaction was allowed to develop at room temperature for 15 min and read at 450 nm.

For analysis of IgG1 binding to F1- or V-antigen peptides, the peptides were added to the plates in 0.05 ml per well at 25 µg/ml. and the plates incubated overnight at 4°C in Immunlon 2HB 96-well plates. After the washing and blocking steps as described above, binding of the human and mouse mAbs IgGs was detected as described above. The binding of all mAbs to the peptides was evaluated at 10 µg/ml.

### Flow cytometry and immunofluorescence analysis of IgG binding to bacterial cells

Flow cytometry was used to analyze the binding of the human anti-F1 and anti-V antigen IgG1s to *Y. pestis pgm-* whole-cells. *Y. pestis pgm-* was first streaked onto a sheep-blood agar plate and grown at room temperature (RT) for 3 to 4 days until colonies were readily visible. A single colony was picked and inoculated into 10 ml of Heart Infusion Broth (Remel, Lenexa, KS) containing 0.2% xylose and 2.5 mM CaCl_2_, and cells grown overnight (o/n) at RT with rigorous shaking. The next day, an aliquot of the bacteria culture was shifted to 37°C, and the other remained at RT, and the cultures were allowed to continue for another 3 hours before the bacteria were collected and suspended in PBS. Ten µL of bacterial cells was mixed with 90 µL of Fc Block solution [PBS with 1% FCS and 10 µg/ml FcBlock (BD Bioscience)] to achieve a final density of 1×10^7^ cells/ml. Human or mouse IgGs were added to the bacterial cell suspension to a final concentration of 10 µg/ml. After incubating at 4°C for 30 min, the bacterial cells were collect by centrifugation and suspended in 1 ml of PBS, and cells washed twice. The bacterial cells were then suspended in the same Fc Block solution, and secondary antibodies, which included either goat anti-mouse IgG-FITC (Pierce, Rockford, IL) or goat anti-human IgG-FITC (Southern Biotech, Birmingham, AL) were added to the cells at a dilution of 1∶100. After 30 min at 4°C, the cells were then washed three times with PBS and subjected immediately to FACS analysis using a FACSCalibur (BD Bioscience, San Diego, CA), after the cells were fixed with 4% paraformaldehyde. For detection of antibody binding to whole-cells by immunofluorescent microscopy, the growth of *Y. pestis pgm-* and the sample preparation for binding by human or mouse anti-F1 or anti-V mAbs was identical as that for the FACS analysis, except a Nikon T-2000 fluorescent microscope (Nikon Instruments Inc., Melville, NY) was used for detection.

### Passive protection by human or mouse anti-F1 or anti-V mAbs and *Y. pestis* challenge studies

Antibodies to be evaluated for their efficacy against a plague challenge were given intraperitoneal (i.p.)(500 µg per mouse, except when stated differently in the figure legend) to 6–10 week old BALB/c mice 24 h before they were challenged or at time points post-challenge as indicated in the figure legend. The challenge dose was prepared from frozen stocks of *Y. pestis* CO92 that were streaked on tryptose blood agar slants and incubated at 28°C for 48 h. After the incubation period, the slants were rinsed with 10 mM potassium phosphate buffer, pH 7.0, and cell density adjusted to the required density with the same buffer. Mice were given the challenge dose of LD_50_ ∼25–40, subcutaneously in 0.2 ml, where 1 LD_50_ is equal to 1.9 cfu [37] and observed for at least 21 days. Research was conducted in compliance with the Animal Welfare Act and other federal statues and regulations relating to animals and experiments involving animals and adheres to principles stated in the *Guide for the Care and Use of Laboratory Animals*, National Research Council, 1996. The facility where this research was conducted is fully accredited by the Association for Assessment and Accreditation of Laboratory Animal Care International. The studies involving mice were approved by the IACUC at the U.S. Army Medical Research Institute of Infectious Diseases, animal protocol number AP-07-040.

### Half-life of human or mouse anti-F1 and anti-V mAbs given passively to mice

An ELISA as described above was used to detect the amount of human or mouse anti-F1 and anti-V mAbs present in mice over time. Briefly, F1-V protein (2 µg/ml in 0.2 M carbonate buffer, pH 9.4)) was used to coat a 96-well plate (Immulon 2HB) overnight at 4°C before washing and blocking. Two-fold dilutions of mouse serum taken retro-orbitally after i.p. administration of the mAb were made in 1X PBS with 1% BSA and 0.05% tween-20, added to plates and incubated for 1 hr at 37°C before washing. The amount of human or mouse anti-F1 or anti-V mAb binding to the antigens was detected by the addition of goat-anti-human or goat-anti-mouse IgG conjugated to HRP (Southern Biotechnology). The results from 3 mice at each time point for each mAb was performed in triplicate and were reported as the mean of the reciprocal of the highest dilution giving a mean OD of at least 0.1, which is at least twice the standard deviation (SD).
